# An Enhanced 3D Model of Intravascular Lymphatic Valves to Assess Leaflet Apposition and Transvalvular Differences in Wall Distensibility

**DOI:** 10.3390/biology12030379

**Published:** 2023-02-27

**Authors:** Christopher D. Bertram, Michael J. Davis

**Affiliations:** 1 School of Mathematics & Statistics, University of Sydney, Sydney, NSW 2006, Australia; 2 Department of Medical Pharmacology & Physiology, University of Missouri, Columbia, MO 65212, USA

**Keywords:** computational model, lymph transport, fluid–structure interaction, finite elements, valve characteristics

## Abstract

**Simple Summary:**

The small size of lymphatic vessels, and thus of their one-way valves, brings problems that have resulted in a unique shape. This study extends a previous computer model of a lymphatic valve to deal with how the valve deforms at the end of closure, when the flexible opposite sides come together to prevent backward flow. The model is also extended to find out the effect of the wall of the vessel in which the valve exists having differing stiffness before and after the valve. In a small accompanying series of experiments, it was found in valves from two out of three locations that a measure of wall stiffness tended to be lower after the valve when the pressure inflating the vessel was low or moderate. The study adds to our knowledge of the behaviour of normal lymphatic valves, potentially aiding identification and management of abnormal behaviour in disease states.

**Abstract:**

Lymphatic valves operate in a fluid-dynamically viscous environment that has little in common with that of cardiac valves, and accordingly have a different, axially lengthened, shape. A previously developed 3D fluid/structure interaction model of a lymphatic valve was extended to allow the simulation of stages of valve closure after the leaflets come together. This required that the numerical leaflet be prevented from passing into space occupied by the similar other leaflet. The resulting large deflections of the leaflet and lesser deflections of the rest of the valve were mapped as functions of the transvalvular pressure. In a second new development, the model was reconstructed to allow the vessel wall to have different material properties on either side of where the valve leaflet inserts into the wall. As part of this, a new pre-processing scheme was devised which allows easier construction of models with modified valve dimensions, and techniques for successfully interfacing the CAD software to the FE software are described. A two-fold change in wall properties either side of the leaflet made relatively little difference to valve operation apart from affecting the degree of sinus distension during valve closure. However, the numerically permitted strains were modest (<14%), and did not allow examination of the large-scale highly nonlinear elastic properties exhibited by real lymphatic vessels. A small series of murine popliteal, mesenteric, and inguinal-axillary lymphatic vessel segments containing a valve were experimentally investigated ex vivo. The pressure–diameter curves measured just upstream and just downstream of the valve were parameterised by computing the difference in tubular distensibility at three values of transmural pressure. In the popliteal and mesenteric segments, it was found that the distensibility was usually greater just downstream, i.e., in the sinus region, than upstream, at low and intermediate transmural pressure. However, there was wide variation in the extent of difference, and possible reasons for this are discussed.

## 1. Introduction

The fundamental problem faced by lymphatic valves, and also by the valves in tiny veins [1], is that at small spatial scales, the liquid flow, whether it be of a dense red cell suspension in blood, or of a dilute suspension of white cells in lymph, is fully viscous. Inertial forces, which for cardiac valves usefully give rise to vortices (recirculating flow), are not available. Consequently, the operation of a lymphatic valve has little in common with that of a cardiac valve beyond the fact that both prevent or minimise back-flow. Anatomically too, while almost all lymphatic valves are bicuspid, like the mitral valve, they do without the elaborate appendages (chordae tendineae, papillary muscles) which that valve utilises to prevent prolapse. Instead, the two leaflets of a lymphatic valve form a funnel having a slit-like exit, with the funnel occupying rather more than one diameter of vessel length. By means of a qualitative mathematical model, Mazzoni et al. [2] explained how this axially extended configuration was the entire key to successful operation in viscous flow. While the valve remained open, but with a developing adverse pressure gradient bringing about flow reversal, the flow back through the funnel incurred an axial pressure drop, while the essentially stagnant fluid outside the funnel, i.e., behind the leaflets, retained the pressure at the funnel entrance. Thus, the viscous backflow brings about a pressure difference across the valve leaflets which shuts the valve. This explanation is necessarily sequential, but of course, the flow-driving effect of pressure differences is instantaneous in the absence of inertia, so all the above steps occur concurrently.

Mechanistically, this insight has scarcely been surpassed today, despite the creation since of many different lymphatic valve models. Many of these have been lumped-parameter models, designed not so much to adduce understanding as simply to provide valve function in a more global lymphatic vessel model. The valve is modelled either as a numerical device which just prevents back-flow [3,4,5,6] or as a resistance to flow that varies with the transvalvular pressure difference [7,8,9]. A cardiac valve model [10] was adapted for lymphatic use by Contarino and Toro [11].

Passing to two dimensions, the flow through such a representation of a lymphatic valve was computed by Macdonald [12]. Two groups have devised two-dimensional models for lymphatic valves [13,14], despite the fact that the lymphatic valve configuration is necessarily and quintessentially three-dimensional. The degree of mechanistic contrivance needed seems at odds with the level of detail that a two-dimensional model implies. However, both models exist simply to provide valve function in a wider lymphatic vascular model.

Thus, to go beyond the mechanistic insight provided by simple qualitative models, it is necessary to model lymphatic valves in three dimensions. Four groups have done this. The first was Wilson et al. [15,16,17], who progressively developed what was originally a rigid model to eventually achieve fully coupled fluid–structure interaction (FSI) between the fluid and the valve leaflets, using ANSYS finite-element software. Also using ANSYS finite-element analysis, Watson et al. [18] applied the transvalvular pressure load to flexible valve leaflets of shape derived from detailed imaging, without considering fluid flow. A 3D finite-element FSI model with all structural parts made flexible was achieved for the first time by Bertram [19]; the model showed how the degree of bias of the valve characteristics to the open position increased with the inter-leaflet gap in the resting position and with valve inflation pressure. A different fluid–structure approach, involving application of the lattice-Boltzmann technique, was taken by Alexeev and Dixon [20,21], after the pioneering work of Buxton and Clarke [22] for vein valves. The method seems to provide unparalleled ability to model leaflets of extreme flexibility.

This paper reports on the further development of the 3D computational model of an intravascular lymphatic valve developed and described by Bertram [19]. In particular the new developments allow investigation of two aspects of valve performance which could not be simulated with the original model. One of these concerns the stage of valve closure which involves the leaflets of the valve coming together. In the original model, no provision was made for this regime. In principle, nothing existed to prevent one leaflet from passing through the plane of symmetry between it and the other leaflet, so that the numerical leaflets would have passed through each other. In practice, the code did not allow this to happen, because it would have changed the topology of the fluid space within the valve. As a result, the model could not proceed past the point where first leaflet contact would have occurred. Provision is now made to recognize and to deal with inter-leaflet contact, or rather contact between the one leaflet explicitly simulated and the plane of symmetry.

The other new development allows the specification of different material properties for the valve wall upstream and downstream of where the leaflet inserts into the valve wall. Because of the existence of the valve, to the extent that the valve is competent, a lymphatic vessel experiences a different cyclic regime of pressure change upstream and downstream of the valve leaflets. It is therefore logical to expect that different material properties might result. Indeed, preliminary studies of mouse lymphatic vessels suggested that pre- and post-valve segments might have different stiffness properties, and so experiments were designed specifically to test this hypothesis. Apart from the observations of Rahbar et al. [23] for rat vessels, the resulting findings below are the only experimental measurements of pre- and post-valve distensibility differences in lymphatics. It is also logical to ask what effects such spatial non-uniformity of wall properties might have on valve performance, and this question can be addressed through modelling. Numerically, the apparently minor change of specifying different properties on either side of the valve forced the development of a whole new way of setting-up and pre-processing the finite-element model. In the belief that the lessons learned can be of use to others, we here detail some aspects of the revised methodology, as well as presenting the final outcome. The question posed above is definitively answered: pre- and post-valve wall distensibility differences do not significantly alter the gating properties of lymphatic valves.

## 2. Methods

### 2.1. Numerical

The original model is described in full by Bertram [19], so only a compressed description is given here. Geometrically, the model mostly followed the form of the idealised valve derived by Wilson et al. [16,17] from measurements on a set of 74 real lymphatic valves from rat mesentery (the two models differed geometrically in the form of the projected orifice between the trailing edges of the valve leaflets.). However, Wilson et al. simulated only the flexible valve leaflet, with the valve wall reduced to a fixed no-slip boundary. In contrast, the Bertram model was flexible throughout, i.e., the tube to which the leaflets attach, including the valve sinus and the uniform tube up- and downstream, was all made flexible. Like the Wilson model, the Bertram model assumed symmetry about two orthogonal planes passing through the valve axis (the *z*-axis), one (*y* = 0) passing between the two leaflets and the other (*x* = 0) cutting both leaflets in half. This allowed only one quarter of the valve to be simulated.

Two-way FSI was specified between the 5 µm thick solid structures of the valve, i.e., the wall and the leaflets, and the fluid contained within, using the capabilities of the finite-element software ADINA (until recently from ADINA R&D Inc., Watertown, MA, USA, now available from Bentley Systems Inc., Exton, PA, USA). The structures and the fluid constituted two separate models within ADINA, interacting at their mutual boundary. Besides the two symmetry boundaries, the fluid model was also bounded by the inlet and outlet faces at which time-varying pressure boundary conditions were imposed: slowly ramped up at inlet to produce forward flow and open the valve, at outlet to produce backflow and close it. At these same furthest upstream and downstream axial locations, the edges of the wall in the solid model were constrained not to move, as for an excised vessel segment tied to cannulae. Four parameters were varied: the stiffness of the neo-Hookean leaflet material, the stiffness of the similarly neo-Hookean wall material, the extent of the initial gap between the trailing edges of the leaflets, and the degree of inflation by uniform pressure before differential pressure was applied. Because the position of the free edge of the leaflet varies greatly near the centre *x* = 0, it was necessary to remesh the finite-element grid for the fluid model (see Figure 2 of Bertram [19]) after a varying number of time- and pressure steps, using both an initial list of times for remeshing and restarts at extra instants when the rate of deformation was high.

For the investigation here of valve closure past the point of first leaflet contact, it was necessary to prescribe contact between that face of the leaflet facing the opposite leaflet and the symmetry plane between the leaflets. In the ADINA FSI scheme, actual contact between these is not allowed, because it would change the topology of the fluid space. Instead a tiny minimum separation between them is enforced, such that the (fictitious) remnant creeping flow through the remaining separation is too small to affect the overall back-flow past the valve. As described in detail by Bertram [19], the physical back-flow occurs because the contact between the trailing edges of the leaflets does not extend all the way to the wall; instead, a small gap persists on each side where the initial geometry prescribed the leaflets exiting the wall at a small angle to the plane between them. First contact between leaflets made identical by the symmetry assumptions necessarily occurs on the leaflet centre-line, with this small remaining gap extending all the way from the wall to the centre (see Figure 10 of Bertram [19]). Although the half-gap progressively reduces to the tiny minimum separation over an increasing distance from the valve axis as closure extends past first contact, it does not disappear, although the remeshed fluid grid becomes somewhat rudimentary in the zone of minimum separation. By running trials with different minimum separations, it was established how small the separation needed to be (0.1 µm between the leaflet and the symmetry plane) that under all adverse pressure differences, the fictitious remnant flow was negligible relative to the physical back-flow through the inter-leaflet gap at the commissure (the most downstream part of the insertion, the C-shaped curve of the join between the leaflet and the valve wall). Since the fictitious back-flow is so small relative to the real back-flow, the fact that the grid for the flow in the zone of minimum separation is not properly resolved is unimportant. All subsequent runs were made under these conditions.

The investigations of closure with leaflet contact otherwise made use of a model that had been constructed using the methods developed originally. These involved first building STL files in Matlab (The Mathworks Inc., Natick, MA, USA) describing triangular surface meshes over the boundaries of the fluid space and of the valve wall excluding the leaflet, then importing these into the fluid and structure modules of ADINA respectively for generation of volumetric meshes filling the volume within each of these two surfaces. For the leaflet, only point coordinates along the bounding surface edges were generated in Matlab; surface and volume creation and meshing were done in ADINA. These methods were laborious, and precluded the easy variation of model dimensions. In addition, the use of imported STL files heavily constrained what model- and mesh-building operations were possible in the ADINA environment.

Accordingly, for the investigation of models involving differing wall properties up- and downstream, a new approach was devised, involving the use of CAD software to construct the 3D shapes of the parts of the valve structure and of the fluid. The chosen software was Solidworks (Dassault Systèmes SolidWorks Corp., Waltham, MA, USA), as used by Wilson et al. [17], able to export Parasolid files, which are the preferred import format for ADINA. Parasolid is proprietary to Siemens Digital Industries Software, but is widely licensed for use by other companies to describe 3D geometric models. In principle, such CAD models are much quicker to construct, involving only a relatively small number of steps to create and meld complex shapes.

**Figure 1:**
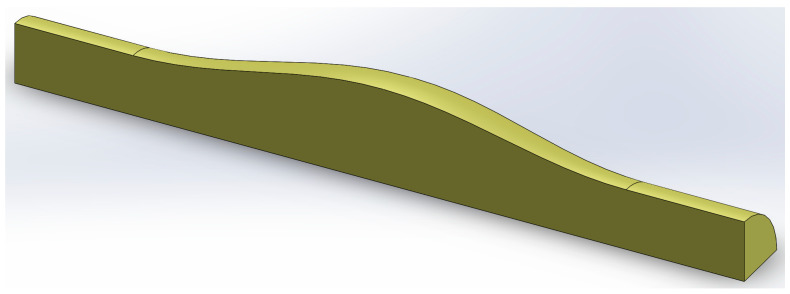
The Solidworks part that was exported via Parasolid and used within ADINA to make the eventual fluid model shape. As explained in the text, the leaflet volume that would eventually be occupied by the leaflet was left in at this stage.

However, in practice, complications arose where individual CAD parts butted up to each other, both within the valve structure (now consisting of three parts: the leaflet, the upstream wall and the downstream wall) and at the interface between the solids and the fluid. These complications were ultimately traceable to rounding errors in Solidworks, relative to the theoretical, equation-defined curves used to construct parts. If CAD parts were constructed according to the actual required dimensions and mathematically correct shapes, the resulting volume models, once in the ADINA environment, were invariably flagged as dimensionally inconsistent. It was found necessary to design the CAD parts with dimensions deliberately varied from the nominal ones, in order that ADINA could be presented with volume models that overlapped into each other. Via Boolean operations within ADINA, the overlap could then be trimmed, leaving volumes that would mesh consistently. Thus, for instance, the fluid model, which over its external surface had a nominal tube inside radius of 50 µm, rising to 80 µm at the top of the valve sinus, was specified with an extra 1 µm of radius everywhere, such that it overlapped into the 5 µm thickness of the valve wall. The extra thickness was then pruned away in ADINA, once the Parasolid files for the up- and downstream wall had been turned into a combined but not merged entity in ADINA that could be used to subtract from the volume occupied by the ADINA fluid model. Similarly, instead of using Solidworks to construct a fluid space that excluded the volume occupied by the leaflet, the leaflet was ignored at this stage, and only once within the ADINA environment was its ADINA realization used to subtract a corresponding shape from the ADINA fluid model; see Figure 1.

**Figure 2:**
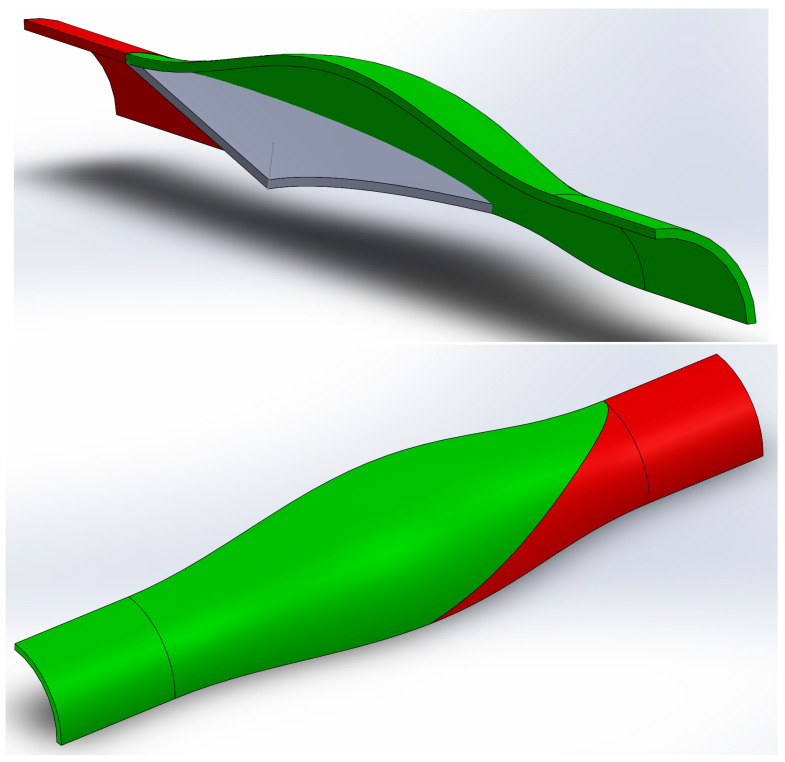
Two views of an assembly in Solidworks of the three parts forming the structure of one-quarter of a valve: the leaflet (grey), and the wall divided into upstream (red) and downstream (green) parts. The assembly here is to nominal dimensions and for illustration only; it was not used for export to ADINA.

A related complexity arose at the interface between the upstream and downstream parts of the wall. Experimentally, it had not been necessary to define exactly where the frontier between these two parts should lie. In the CAD environment, it rapidly became clear that the only logical place to effect this division was at the insertion; see Figure 2. Because of the peculiar shape of these low-Reynolds-number valves, the insertion is a three-dimensional curve which can be projected radially through the wall, thereby forming a cutting surface. Given that the leaflet has finite thickness and, particularly near its centre-line joins the wall at an acute angle, there are actually two such curves: at the upstream or downstream extremities of the join, forming apparently equally meritorious alternatives. However, in practice, whereas the upstream curve was defined by an analytical geometrical equation used in the Solidworks model construction, the downstream curve simply emerged as a result of the parts intersecting, and was thus not available for radial projection.

However, just as it had been necessary to leave the cutout of the leaflet shape and the excess fluid radius to ADINA, so it was necessary (a) to let one of the two ADINA wall parts cut off part of the other where they met, and (b) to situate the meeting place between the wall parts slightly away from the leaflet (ultimately, 1 µm upstream of the upstream edge of the insertion), so that a single ADINA wall part (the downstream one) could be used to cut excess off the Solidworks-specified dimensions of the ADINA leaflet volume. So, in Solidworks, the leaflet part was bounded where it was ultimately to meet the valve wall by simply projecting normal to the valve face (in the direction shown in the upper panel of Figure 2 by a short line projecting from the leaflet surface) a cutting curve which was the inside, i.e., most upstream, extremity of the ultimate leaflet-to-wall insertion. This created a part which, as depicted in Solidworks, was uniformly thick everywhere, with no bevelling at the insertion, and projected right through the valve wall slightly into the external surroundings. The discovery of all these expedients took much time.

### 2.2. Experimental

**Figure 3:**
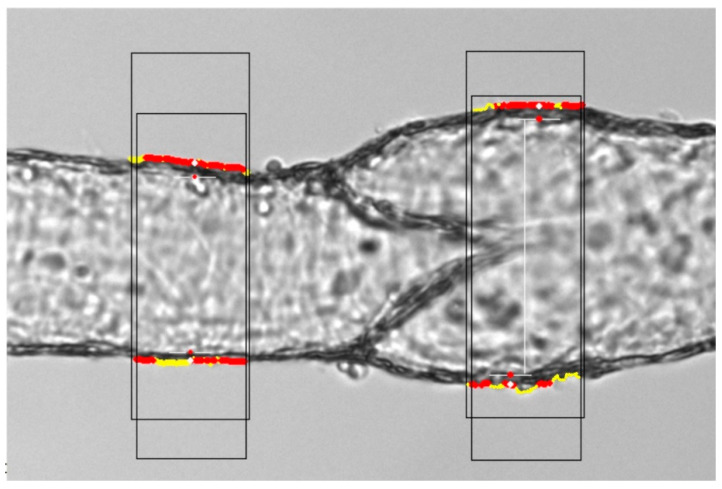
Brightfield image showing by overlay of the gates using for tracking diameter where the upstream and downstream sites were located relative to the valve (20AugV1.mes).

The biological methods are essentially those described in detail by Davis et al. [24]; only a summary will be given here. Lymphatic vessel segments dissected from anaesthetised mice were mounted on glass micropipettes in a bath of warm physiological saline under an inverted microscope. The bath solution was calcium-free and included the calcium-selective chelating agent EGTA, to block any possibility of spontaneous lymphatic muscle contractions during the measurements. The pipettes led to reservoirs of similar physiological perfusate in which the pressure was computer-controlled in a servo feedback loop. Video of the vessel segment from a camera mounted on the microscope was recorded synchronously with the digitized pressure signals and a continuous diameter measurement at a chosen site along the vessel by the tracking method of Davis [25]. Subsequently offline, diameter traces were tracked from the video at other sites than the one monitored during the experiment. Figure 3 shows an example of where the tracking sites were located, just upstream and downstream of where the insertion met the wall at the base of the valve. Eight single-valve segments from mesenteric lymphatics were so treated, plus four from popliteal vessels and four from inguinal-axillary vessels.

In the procedure leading to the traces analysed here, the pressure in both reservoirs was raised in a linear ramp from 0.5 to 10 cmH_2_O over the course of 1 min. The depth of saline immersion was 5 mm, so the transmural pressure *Δp* applied to the segments was 0.5 cmH_2_O less than the distending pressure (the joint reservoir pressure). The ramp data of diameter vs. transmural pressure were least-squares fitted by the equation

*D* = *c*_1_ + *c*_2_[1 − exp(−*Δp*/*c*_3_)] + *c*_4_.*Δp* (1)

then the vessel distensibility **D** was calculated as

**D** = (*dD*/*dΔp*)/*D* = [*c*_2_.exp(−*Δp*/*c*_3_)/*c*_3_ + *c*_4_]/*D* (2)

Strictly, **D** = (*dA*/*dΔp*)/*A* = 2(*dD*/*dΔp*)/*D*, *A* being the cross-sectional area π*D*^2^/4, but for this comparative study, the factor of two is ignored.

## 3. Results

### 3.1. Experimental

**Figure 4:**
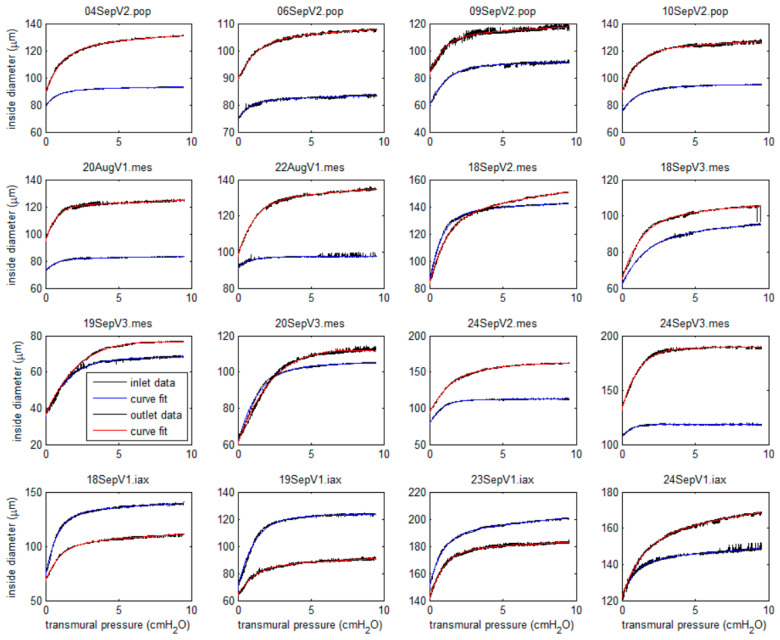
Distensibility of the vessel at inlet (blue) and outlet (red) of a valve. For each of the 16 mouse experiments shown, data of transmural pressure vs. diameter (black) are shown overlaid by the fitted curve: inlet (blue) and outlet (red). Valves from four popliteal (pop), eight mesenteric (mes), and four inguinal-axillary (iax) lymphatic vessels.

The raw data of diameter vs. transmural pressure at each of the two sites of measurement, just upstream and just downstream of where the valve leaflets were visible in the backlit video record, are presented in Figure 4 (black traces), overlaid by the fitted curves according to Equation (1) (blue for the upstream site, red for the downstream one).

In eleven cases, the diameter was greater for a given pressure just downstream of the valve over the whole of the pressure/diameter relation. In one mesenteric segment, the diameter upstream slightly exceeded that downstream at all transmural pressures from zero to almost 3.5 cmH_2_O; only at pressures equal to or greater than 3.5 cmH_2_O did diameter downstream exceed diameter upstream for the same pressure. Another mesenteric vessel displayed this property but with cross-over at 2.5 cmH_2_O. The valve segments from inguinal-axillary vessels tended to behave differently; in three out of four cases, diameter upstream was greater than diameter downstream at every pressure. In these vessels, there was no real valve sinus at all; the valve often occurred in a smaller part of a vessel that varied in diameter slightly along its whole length.

The Cauchy or engineering strain at *Δp* = 9.5 cmH_2_O varied widely; the maximum was 110% at the outlet of specimen 9 (19SepV3.mes), and the minimum was 6.9% at the inlet of specimen 6 (22AugV1.mes). Over all specimens, the mean inlet strain at *Δp* = 9.5 cmH_2_O was 43.7% ± 7.6% (s.e.m.); the mean outlet strain was 53.1% ± 6.1%. By a Behrens-Fisher two-tailed *t*-test, these means did not differ significantly.

**Figure 5:**
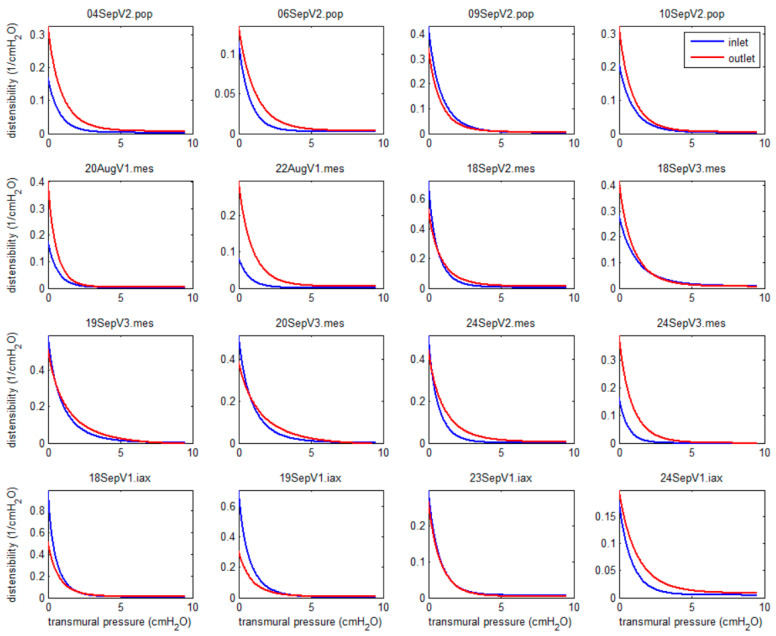
The distensibility of the valved segments of lymphatic vessel, at a site just upstream of the valve (blue) and another just downstream (red).

Because the passive elastic properties of lymphatic vessels are so strongly nonlinear, it is impossible to summarise the stiffness or inverse stiffness of a segment by a single number; one must consider the entire curve of distensibility vs. pressure, as presented in Figure 5. In each case, the distensibility curves computed according to Equation (2) from the curves fitted to the data are shown (blue for the upstream site, red for the downstream one).

**Figure 6:**
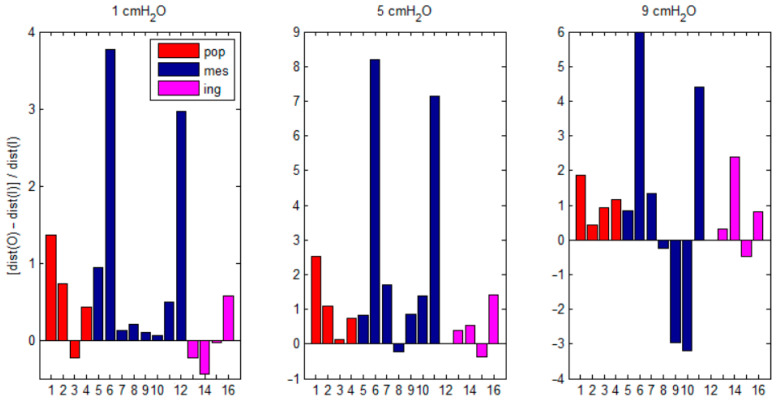
Normalised distensibility difference at 1, 5, and 9 cmH_2_O of transmural pressure, for all 16 measured valves: popliteal (red), mesenteric (blue) and inguinal-axillary (magenta). For segment 12 (24SepV3.mes), values at 5 and 9 cmH_2_O are omitted because **D**_O_ and **D**_I_ were both very close to zero.

In essentially all cases analysed, the distensibility fell quasi-exponentially with increased distending pressure. (In one case, 20SepV3.mes, the pressure/diameter relation showed slight reverse curvature; as the pressure increased, the slope *dD*/*dΔp* was less at the lowest *Δp* than at intermediate values. This case could be marginally better fitted by a modified logistic equation *D* = *c*_1_ + *c*_2_/{1 + exp[−*c*_3_/(*Δp* − *c*_4_)]} + *c*_5_.*Δp*, giving rise to a peak of distensibility for both inlet and outlet between 0.5 and 1 cmH_2_O, not shown here.) The distensibility was higher downstream than upstream at all pressures in seven segments; in the others, the distensibility curves crossed over once or more. To summarise the results more compactly, a curve of normalised distensibility difference between the two sites vs. transmural pressure was computed as *N*(*Δp*) = [**D**_O_(*Δp*) − **D**_I_(*Δp*)]/**D**_I_(*Δp*), where suffix I denotes the valve inlet and O the outlet. *N* takes values greater than zero at values of *Δp* where **D**_O_ > **D**_I_, and vice versa; it is zero where **D**_O_ = **D**_I_. Figure 6 presents this normalised distensibility difference for all 16 segments, at three selected values of transmural pressure: 1, 5, and 9 cmH_2_O.

### 3.2. Numerical

#### 3.2.1. Valve Closure

**Figure 7:**
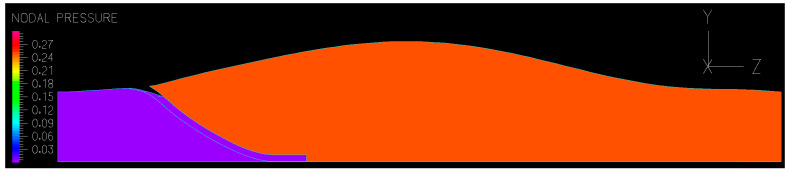
Fluid pressure at the plane *x* = 0 at maximal closure. The leaflet trailing edge lies along the plane *y* = 0. The units of the pressure scale are kPa (1 kPa = 10^4^ dyn/cm^2^).

**Figure 8:**
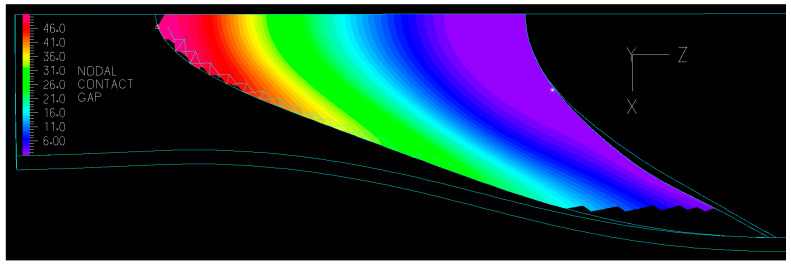
The half-gap under the leaflet at maximal closure. The nominal minimum was set at 0.1 µm. The distance scale is in µm.

**Figure 9:**
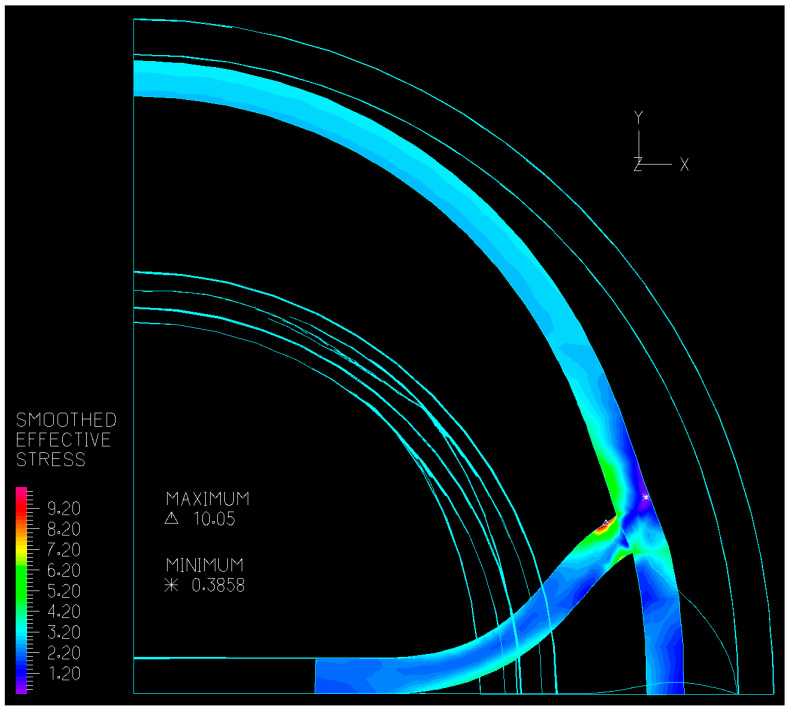
A sectional view of the magnitude of stress induced by bending in the valve structure at maximal closure. Section at the axial position of the leaflet trailing edge at *x* = 0 in the initial unstressed condition, with other salient sections projected into the plane. The coloured stress contours do not extend all the way across the leaflet, because the central part of the leaflet has been pushed upstream of this location. At lower right can be seen the small remaining gap responsible for the leakage flow-rate. The scale is in kPa.

**Figure 10:**
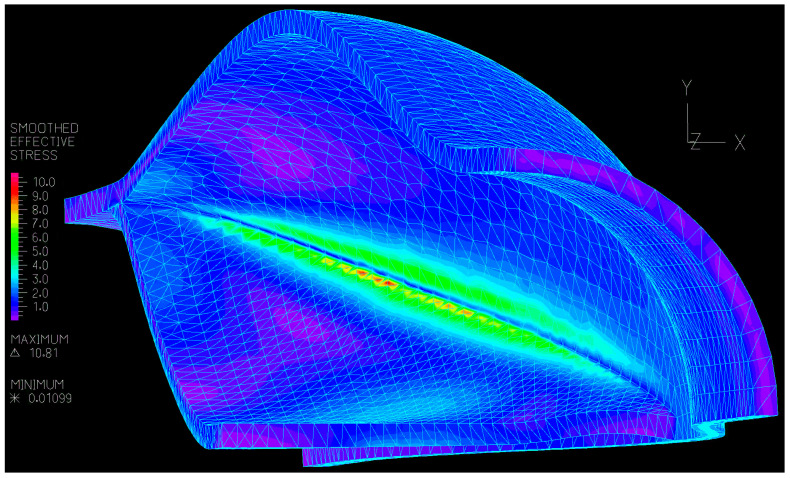
Another view of the stress in the valve structure, showing how it is concentrated in the region of the leaflet insertion, manifesting in both the leaflet and the valve wall. The scale is in kPa.

Valve closure was investigated in a model with wall shear modulus uniformly 20 kPa and leaflet shear modulus 10 kPa. Figure 7, Figure 8, Figure 9 and Figure 10 show aspects of the valve configuration when it is maximally closed. Comparison between the leaflet positions in Figure 2 and Figure 7 shows how much the leaflet has been deflected by the adverse pressure difference of 2500 dyn/cm^2^ at this maximal closure state. With only minimal leakage flow-rate through the remaining side-passage, the pressure in the fluid is essentially uniform on each side of the leaflet.

The separation at each (*x*,*z*)-coordinate between the inside of the leaflet and the symmetry plane *y* = 0 (half of the gap between the two leaflets) is shown by means of contours in Figure 8. The zone of the lowest (purple) contour shows the area of the leaflet that is essentially in apposition, extending substantially back from the trailing edge on the centre-line, but then progressively narrowing towards the leaflet commissure.

The stress in the valve wall and in the leaflet is shown in two views in Figure 9 and Figure 10. Figure 9 shows a cut through the valve at the axial position which is tangent to the trailing edge of the leaflet on the centre-line in the initial state. The leaflet, deflected away from its rest position down towards the symmetry plane *y* = 0, causes substantial bending in the tube wall, and for both parts of the structure, the greatest stress concentration occurs where the leaflet and the wall join. However, the least stress magnitude in this section occurs in the outermost layer of the wall, immediately adjacent to where the leaflet joins the inner wall. Elsewhere, the leaflet is also exposed to substantial stress where it bends down to meet the symmetry plane. The view also shows in outline the tube wall at each end of the model, and the underside of the leaflet where it meets the tube wall. At this point, there is a narrow gap between the modelled leaflet and its symmetrical opposite number, through which courses the back-flow which causes the resistance of the valve to remain finite at this adverse pressure difference. It should be remembered that this gap is here seen in projection; the leaflet trailing edge continuously varies in *z*-location as seen in Figure 8, so the gap is actually a more complicated three-dimensional shape than is obvious here. The reason that the gap exists is that in the valve at rest, the leaflets meet the wall at a small angle from 90°, and the valve is thus partly open at rest (see Figure 3 of Bertram [19]). This bias to the open position, which appears characteristic of lymphatic valves, means that a finite back-flow, propelled by a corresponding adverse pressure difference, must exist before the valve can close.

The oblique view looking upstream which forms Figure 10 shows the several locations where stress is least when the valve is in this maximally closed position. These include the leaflet itself where it lies flat against its opposite number, or in this simulation, against the symmetry plane. Again, the large stress concentration in the leaflet upper surface next to where it meets the wall is evident, but this concentration does not extend as far as either the commissure or the base of the valve (defined as the most upstream part of the insertion). Whilst the greatest stress occurs in the leaflet, very substantial stress is engendered in the wall as well.

#### 3.2.2. Wall Stiffness Change

**Figure 11:**
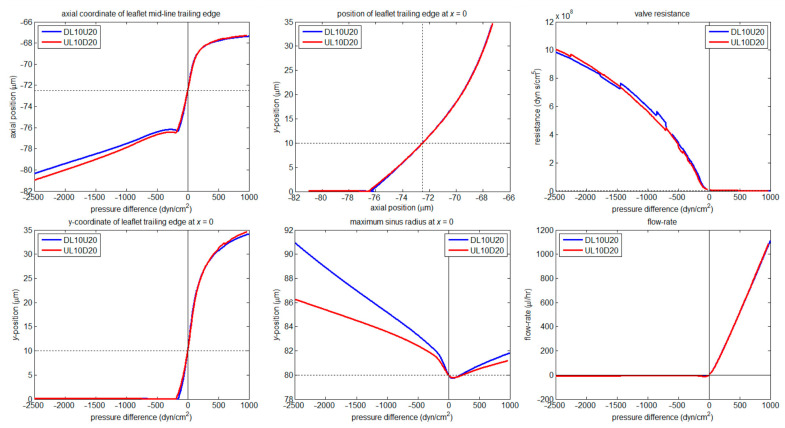
Response of the simulated valve to pressure ramps. Positive pressure differences result from ramping up the upstream pressure, thereby opening the valve, negative ones from ramping up the downstream pressure to close it. Blue curves: wall shear modulus is 20 kPa upstream, 10 kPa downstream. Red curves: wall shear modulus is 10 kPa upstream, 20 kPa downstream.

For these simulations the shear modulus of the leaflet material was 10 kPa throughout. That of the wall material was either 10 kPa upstream and 20 kPa downstream, or vice versa. See Figure 11, which compares salient characteristics of the two valves’ performance in six panels. Four of these are in formats presented by Bertram [19]: the deflection in the *y*-direction of the trailing edge of the leaflet on the centre-line *x* = 0 (lower left), the maximum radius of the sinus on the centre-line *x* = 0 (lower centre), the flow-rate through the valve (lower right) and the valve resistance to flow (upper right), all as functions of the applied pressure difference. The remaining panels show the variation in axial position (*z*-coordinate) of the trailing edge of the leaflet on the centre-line vs. pressure difference (upper left), and, by combining information from the upper left and lower left panels, the variation in two-dimensional (*y*,*z*) position of this same point (upper centre). This last shows how the leaflet swings down as the valve closes, but is then prevented from further deflection by its opposite number if symmetry holds.

Starting at the lower left, the initial (zero pressure difference) position of the *y*-coordinate of the trailing edge of the leaflet on the valve centre-line is 10 µm, this being the resting half-gap specified for this model. All the region where the *y*-coordinate is very close to zero (pressure difference < −157 dyn/cm^2^ for the blue curve, <−190 dyn/cm^2^ for the red curve) is new, because this is the region which Bertram [19] did not compute, where further leaflet deflection at the centre is prevented by contact. The panel above this one shows that after first contact, the trailing-edge centre-line point actually moves slightly downstream initially as contact changes the overall form of the leaflet deflection, then it moves progressively upstream as the leaflet bends more and more under the increasing load of the adverse pressure difference. This upstream movement is slightly greater for the valve with less stiff wall material upstream, despite the fact that the leaflet was then everywhere attached to the stiffer wall material; the reason is that the more compliant wall upstream allows slightly more valve distension upstream, in the process rotating the leaflet insertion slightly clockwise in the plane of Figure 7 and giving the leaflet slightly more space to swing down and upstream.

The overall message of Figure 11 is that whether the valve wall material is stiffer upstream or downstream, there is effectively no difference in valve function, most fundamentally shown in the plot of flow-rate vs. pressure difference (lower right panel). When the valve is closed, the maximum sinus radius varies substantially from blue to red (lower middle panel), because the sinus radius is almost entirely a function of the downstream wall properties alone, but in all other respects, the differences between the two valves are small, and in the case of the valve resistance (upper right panel) in the region involving leaflet contact, small enough to be within the range of uncertainty resulting from jogs in the curve which occurred when the fluid space was remeshed. Such numerical factors necessarily show up here because valve resistance in the closed-valve region is the result of dividing a large number (the adverse pressure difference) by an extremely small one (the leakage flow-rate). In fact, the valve resistance is here tracked to a value of 10^9^ dyn s/cm^5^, an order of magnitude higher than was possible without accounting for leaflet contact.

**Figure 12:**
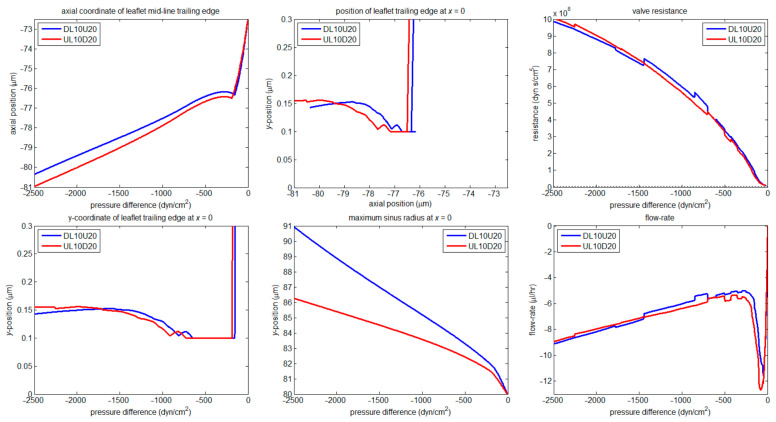
As Figure 11 but with axes arranged to magnify the behaviour during valve closure.

At the cost of making the numerical discontinuities associated with remeshing more evident, Figure 12 presents the same quantities as in Figure 11, but with the axes arranged to magnify regions of special interest. The small regurgitant flow-rate passes through a maximum well before the leaflets reach apposition, then a minimum around apposition, before increasing again slowly with further-increasing negative transvalvular pressure difference. This final flow-rate increase happens because the bending stiffness of the now strongly pushed-together leaflets resists further diminution of the small tear-drop-shaped spaces between them at the downstream and most lateral parts of the insertion (see Figure 10 of Bertram [19]). For the same reason the valve resistance increases more slowly at the largest negative transvalvular pressure differences. The half-gap between the trailing edges of the leaflets on the leaflet centre-line, having reached the prescribed minimum of 0.1 um, then increases again slightly (lower left panel) as the centre-line contact between them changes from being at a point to being along a line. The tiny axial regurgitant flow through this microscopic gap (which remains negligible relative to the back-flow through the ‘tear-drop’ side gap) brings a concomitant axial pressure drop. With the downstream pressure for this flow effectively fixed by the pressure boundary condition applied at the valve inlet, the axial pressure drop must increase the pressure between the trailing edges of the leaflets, pushing them apart again slightly, despite the continuing large adverse pressure difference.

**Figure 13:**
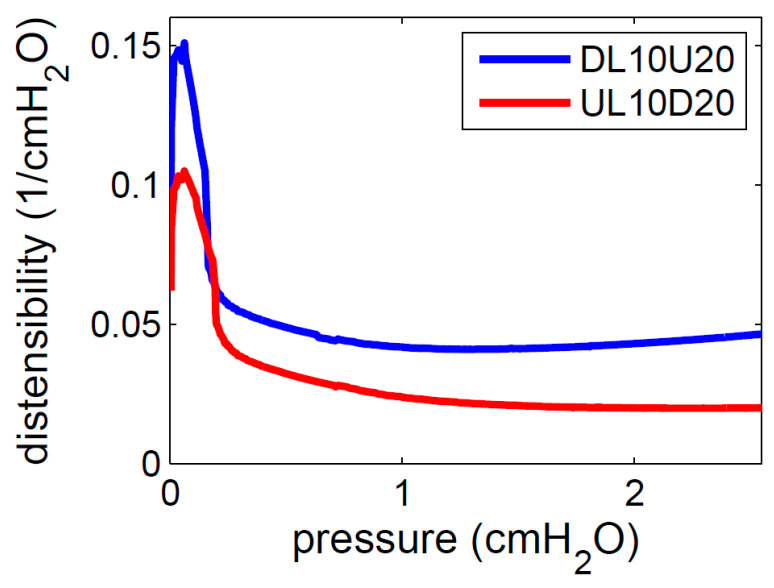
The distensibility of the model valve sinus at its maximum extent, computed from data shown in Figure 12, but expressed in the units of the experiments (1 cmH_2_O = 981 dyn/cm^2^). Blue curve: wall shear modulus is 20 kPa upstream, 10 kPa downstream. Red curve: wall shear modulus is 10 kPa upstream, 20 kPa downstream.

Figure 7 shows that when the valve is closed, the whole of the positive pressure applied at the valve outlet is exerted on the sinus. On this basis, one can make a link between the numerical model and the experiments by calculating an approximate distensibility (*dD*/*dΔp*)/*D* for the sinus from the data shown in the lower middle panel of Figure 12. Here, *Δp* is taken to be the outlet pressure, since the external pressure is zero, and *D* is twice the *y*-position shown, although the factor of 2 cancels. The result is shown in Figure 13. Prior to the valve closing fully, not all of the pressure applied at the outlet is exerted as distending pressure, so the initial upstroke in distensibility very close to zero pressure should be disregarded, but once the flow-rate becomes small, the error is also small. The distensibility is also approximate because the sinus at its maximum extent is not totally circular, being slightly distorted by the leaflet insertions, but this error applies equally to the experiments. Similarly, the higher distensibility at low pressures may in part reflect slight changes in circumferential shape not apparent from a single measure of ‘radius’ in one azimuthal direction, but again this could occur also for the experiments. Comparison of Figure 5 and Figure 13 shows that the maximum distensibility of real valve wall at low transmural pressure, while varying greatly from one specimen to another, could be five or more times greater than that in the model, while the experiments explored distending pressures almost four times greater.

## 4. Discussion

The model’s assumption of symmetry about the orthogonal planes *x* = 0 and *y* = 0 brings both advantages and disadvantages. The obvious advantage is the reduction in the number of computational cells, which may either reduce the time taken or bring a given level of grid fineness within the capability of the computing resources in use. This is especially important when the fluid–structure interaction is being solved monolithically as here, i.e., by inversion at each step of a single very large sparse matrix, rather than iteratively by solving alternately the fluid problem and the solid problem, which has grave ramifications for stability and convergence [26]. However, it is unlikely that any real lymphatic valves are perfectly symmetrical, if only as a result of uneven constraint of the lymphatic vessel locally by surrounding tissue. Slight asymmetry would in general be expected to reduce the resistance of the leaflets to being deformed into the closed configuration, because it would allow access to extra degrees of freedom. Gross asymmetry would hazard the effective operation of the valve altogether. Although asymmetrical valves are often perceived experimentally (see, e.g., the imaging by Watson et al. [18]), there are no data on which to found a systematic investigation of such configurations at this time.

The recorded pressure/diameter data have here been presented both in their own right and as fitted curves of the true distensibility (*dp*/*dD*)/*D*, thereby circumventing the difficulty of differentiating necessarily noisy experimental data. Distensibility is a property of the whole vessel locally; it is not possible to relate distensibility to actual valve wall material properties in the absence of measurements of vessel wall thickness. Although such measurements can be made, their use to calculate material properties would be notional only, because it is highly unlikely that the whole thickness of this microscopic structure contributes equally to resisting stretch.

Even relative to small blood vessels, lymphatic vessels exhibit a notoriously high degree of elastic non-linearity [23], and it has been suggested [27] that this characteristic minimises oedema, whether arising from increased capillary filtration or from venous hypertension. The non-linearity manifests as a very dramatic decline, of up to two orders of magnitude, in the distensibility of the vessel as increasing internal pressure causes increasing stretch. This greatly complicates the task of deciding whether there is a difference in distensibility from one side to the other of the valve, since the curves of inlet and outlet distensibility cross over in some cases, and sometimes more than once as pressure increases (Figure 5). For three of the four popliteal valves, the distensibility was greater on the outlet side at all three *Δp*-values shown in Figure 6. At 1 cmH_2_O, this was true also for all the mesenteric valves, but at higher *Δp*-values, this was not always the case; by 9 cmH_2_O, the distensibility was greater on the inlet side in three out of seven cases. The greatest inlet/outlet differences in normalised distensibility occurred at intermediate *Δp*, but not invariably. For the three inguinal-axillary valves with smaller diameter at outlet, the distensibility was greater at the inlet of this valve at 1 cmH_2_O, but only in one valve was this so at all three *Δp*-values. Overall, insofar as conclusions can be drawn from measurements on just sixteen specimens drawn from three different types of mouse lymphatic vessel, there is a clear trend that the distensibility of popliteal and mesenteric vessels is greater just after the valve, at least at low and intermediate transmural pressure. This may possibly relate to reduced coverage with lymphatic muscle cells of the sinus region, as observed by Petrova et al. [28], and also in a mouse model of hypercholesterolemia by Davis et al. [29].

As an aside, whether or not the sinus is more extensible has also been questioned for venous valves. Buxton and Clarke [22] stated that "the sinus regions of the vein are more distensible than other regions of the vein wall", apparently based on words of Lurie et al. [30]: “The combination of easily extensible wall of the sinus and stiff material of the valve cusps …”, citing Ackroyd et al. [31] to back up this statement. However, Ackroyd et al. found only that “the ultimate tensile strength of valve leaflet was … twice that of vein wall”, but UTS concerns breaking stress, which is not relevant to extensibility. Ackroyd et al. also reported that “there were small but significant differences between the unpreserved strips of wall and the valve leaflets, the latter being the more extensible”. Thus, Lurie et al. inverted the truth.

The present small study has limitations. The properties of the lymphatic tissues making up a vessel in the immediate surrounds of a valve could vary locally spatially in complex ways. Whether one of the outcomes is a measurable difference in hoop distensibility between the vessel just before the leaflet insertions and at peak sinus is clearly a crude way to approach this reality. Even in the absence of biological variation from vessel to vessel, or valve to valve, the findings might be expected to depend sensitively on exactly where measurements are made axially in relation to the leaflets, to an extent that inevitable experimental variation in positioning could confound the outcomes. (However, when we performed retracking tests, the retracked curves were very similar to the original ones, so this issue may not be as significant as one might anticipate.) It should also be noted that hydrostatic pressure gradients are minimal for the mouse. The lymphatic valves in the leg of a bipedal human have to deal with large hydrostatic gradients, breaking up what would otherwise be a liquid column of 1.5 m height into discrete segments such that no one valve or lymphatic vessel is exposed to the whole of the distending pressure that such a column would represent [32]. Thus, it may be that valves in human leg lymphatics develop much more significant differences in wall distensibility across the valve than evidenced here, but the matter has not been investigated. The question has been posed for rat mesenteric lymphatics, where Rahbar et al. [23] found that the passive pressure–diameter response did not vary between mid-lymphangion and valve regions, similarly to what we found in mouse inguinal-axillary.

In contrast to the large strain range seen in the ex vivo measurements, the 3D valve model is confined to modest strains; a maximum circumferential tensile strain of approximately (91 − 80)/80 = 13.75% is produced in the sinus at maximal closure for the valve with less stiff material downstream (Figure 12). The distensibility of the model sinus fell by a factor of 3.7 (Figure 13, blue curve, less stiff sinus) or 5.2 (red, stiffer sinus) as the distending pressure increased to 1–2 cmH_2_O, but these decreases are small relative to those exhibited by the valve specimens (Figure 5). The neo-Hookean material assumed in the model essentially models elastin [17], whereas the high nonlinearity of the biological wall strain response (Figure 4) reflects the influence also of collagen fibres. However, most of that influence is exerted at higher strains than were reached in the model. Valve closure, while dramatic in how far the valve leaflet moves from its rest position, is achieved with little tensile or compressive strain throughout the thickness of any part of the valve; however, the bending of the leaflet creates significant adjacent tensile and compressive stresses on opposite sides of the leaflet thickness (Figure 9).

At maximum closure, the greatest stresses due to bending are induced on either side of the leaflet insertion, i.e., in both the valve wall and the leaflet, in a broad region extending around much of the C-shape of the insertion, but excluding the commissures and the base of the valve (middle of the leaflet). It is likely that the simulation exaggerates the extent to which the insertion zone really experiences stresses greatly in excess of those occurring elsewhere in the valve structure. Obviously, the sharp corners between the leaflet and the wall visible in Figure 9, responsible for stress concentration, would in biological reality be absent. Moreover, whereas the material of the simulated leaflet is everywhere of uniform stiffness, as is that of the wall in the closure simulation, the biological insertion may consist of more robust material than that in the rest of leaflet and the valve wall. Finally, the leaflet thickness may increase approaching the insertion, again something not modelled here. Spatially varying leaflet stiffness was modelled by Buxton and Clarke [22] for vein valves and by Watson et al. [18] and Li et al. [13] for lymphatic valves.

The results shown in Figure 11 make clear that, at least for the idealised valve configuration defined by Wilson et al. [16], it makes very little difference to the operation of the valve whether the valve wall material is stiffer upstream or downstream. Only one major difference is seen, in the extent to which the valve sinus balloons when the valve is closed and a large distending pressure builds up downstream. There is a small but distinct difference in how far upstream the centre-line trailing edge of the leaflet gets displaced during closure by a given adverse pressure difference: slightly further with less stiff material forming the upstream part of the valve wall. However, no significant difference in valve resistance to flow at any pressure difference, adverse or favourable, is created by swapping the stiffnesses of the two valve wall parts. Again, it is emphasized that this conclusion is derived from simulations which did not involve major changes in the valve dimensions. The very high distensibility of valve wall at low transmural pressures, for some but not all valves (as high as 0.8 /cmH_2_O in one case), as revealed by the ex vivo experiments, suggests that much greater dimensional changes could occur in vivo, in response to changing pressure inside or outside the lymphatic vessel containing the valve. By changing the degree of overlap between the leaflets, such events could effect much more alteration in valve characteristics, including, at the extreme, incompetence if the leaflets do not completely overlap.

## 5. Conclusions

The paper has addressed a notable deficiency of the numerical model of Bertram [19], namely that it could not continue past the point of first contact between the leaflets as the valve closed. The results showed in detail the shapes adopted by the leaflets as closure progresses, culminating in a configuration where the two leaflets are pressed together at their downstream trailing edges across the whole of the sinus diameter except for very small slits which survive at the commissures.

In a separate development, the model was also reconstructed to allow different wall material properties to be specified before and after the leaflet insertion. This allowed the model to be used to investigate whether a significant difference in vessel wall stiffness upstream and downstream would affect the operating characteristics of the valve itself. Because a competent valve causes a different temporal mean pressure within the lymphatic vessel on either side of the valve when the vessel experiences an adverse pressure difference between its ends, there is reason to expect that different wall properties might result. This hypothesis was investigated experimentally in lymphatic valves from three locations in the mouse, and, although the study was too small for definitive conclusions to be drawn, there was a strong tendency for higher distensibility after the valve at low and moderate pressures for specimens from two of the three locations. However, when a wall stiffness difference amounting to a factor of 2× was investigated numerically, reversing the wall stiffness difference made essentially no difference to the valve operating characteristics.

This combined numerical and experimental study has thus added to our knowledge of the mechanical behaviour of normal intravascular lymphatic valves, potentially aiding identification and management of abnormal behaviour in disease states.

## Data Availability

The data presented in this study are available upon reasonable request from the corresponding author.
